# Supplementary material for: Next-Generation Computationally Designed Influenza Hemagglutinin Vaccines Protect against H5Nx Virus Infections
Source: Pathogens. 2021 Oct 20;10(11):1352. doi: 10.3390/pathogens10111352 (PMC8625041; doi:10.3390/pathogens10111352)
Supplement: Supplementary file 1 [file pathogens-10-01352-s001.zip › pathogens-1388995-supplementary.pdf]

Supplementary Materials

IAN1

MEKIVLLLAVVSLVKSDQICIGYHANNSTEQVDTIMEKNVTVTHAQDILEKTHNGKLCDLNGVKPLILKDCSVAGWLLGNPMCDEFLNV
PEWSYIVEKANPANGLCYPGNFNDYEELKHLLSRINHFEKIQIIPKSSWSNHEASSGVSSACPYQGRSSFFRNVVWLIKKNNAYPTIKR
SYNNTNQEDLLILWGIHHPNDAAEQTKLYQNPTTYVSVGTSTLNQRLVPKIATRSKVNGQSGRMEFFWTILKPNDAINFESNGNFIAPE
YAYKIVKKGDSAIMKSELEYGNCNTKCQTPIGAINSSMPFHNIHPLTIGECPKYVKSNKLVLATGLRNSPQRERRRKRGLFGAIAGFIEG
GWQGMVDGWYGYHHSNEQGSGYAADKESTQKAIDGVTNKVNSIIDKMNTQFEAVGREFNNLERRIENLNKKMEDGFLDVWTYNAE
LLVLMENERTLDFHDSNVKNLYDKVRLQLRDNAKELGNGCFEFYHKCDNECMESVRNGTYDYPQYSEEARLKREEISGVKLESIGTY
QILSIYSTVASSLALAIMVAGLSLWMCSNGSLQCRICI

IAN2

MEKIVLLLAIVSLVKSDQICIGYHANNSTEQVDTIMEKNVTVTHAQDILEKTHNGKLCSLNGVKPLILKDCSVAGWLLGNPMCDEFLNVP
EWSYIVEKDNPANGLCYPGNFNDYEELKHLLSSTNHFEKIQIIPRSSWSNHDASSGVSSACPYNGRSSFFRNVVWLIKKNNAYPTIKR
SYNNTNQEDLLILWGIHHPNDAAEQTKLYQNPTTYVSVGTSTLNQRSVPEIATRPKVNGQSGRMEFFWTILKPNDAINFESNGNFIAP
EYAYKIVKKGDSAIMKSELEYGNCNTKCQTPMGAINSSMPFHNIHPLTIGECPKYVKSBKLVLATGLRNKPQNSPQRERRRKRGLFGA
IAGFIEGGWQGMVDGWYGYHHSNEQGSGYAADKESTQKAIDGVTNKVNSIIDKMNTQFEAVGREFNNLERRIENLNKKMEDGFLDV
WTYNAELLVLMENERTLDFHDSNVKNLYDKVRLQLRDNAKELGNGCFEFYHKCDNECMESVRNGTYDYPQYSEEARLKREEISGVK
LESIGTYQILSIYSTVASSLALAIMVAGLSLWMCSNGSLQCRICI

IAN3

MEKIVLPFAVISLVKSDQICIGYHANNSTEQVDTIMEKNVTVTHAQDILEKTHNGKLCDLNGVKPLILKDCSVAGWLLGNPMCDEFIRVP
EWSYIVERANPANDLCYPGNLNDYEELKHLLSRINHFEKTLIIPKSSWPNHETSLGVSAACPYQGTPSFFRNVVWLIKKNDAYPTIKISY
NNTNREDLLILWGIHHSNNAAEQTNLYKNPTTYVSVGTSTLNQRLVPKIATRSQVNGQRGRMDFFWTILKPNDAIHFESNGNFIAPEY
AYKIVKKGDSTIMKSEVEYGHCNTKCQTPIGAINSSMPFHNIHPLTIGECPKYVKSNKLVLATGLRNSPLNVPQRETRGLFGAIAGFIEG
GWQGMVDGWYGYHHSNEQGSGYAADKESTQKAIDGITNKVNSIIDKMNTQFEAVGKEFNNLERRIENLNKKMEDGFLDVWTYNAEL
LVLMENERTLDFHDSNVKNLYDKVRLQLRDNAKELGNGCFEFYHKCDNECMESVRNGTYDYPQYSEEARLNREEISGVKLESMGTY
QILSIYSTVASSLALAIMIAGLSFWMCSNGSLQCRICI

IAN4

MEKIVLLFAIVSLVKSDQICIGYHANNSTEQVDTIMEKNVTVTHAQDILEKTHNGKLCDLNGVKPLILKDCSVAGWLLGNPMCDEFINVP
EWSYIVEKANPANDLCYPGNFNDYEELKHLLSRINHFEKIQIIPKDSWSDHEASLGVSAACPYQGNSSFFRNVVWLIKKNDAYPTIKKS
YNNTNREDLLVLWGIHHPNDAAEQTRLYQNPTTYISVGTSTLNQRLVPKIATRSKVNGQSGRMDFFWTILKPNDAIHFESNGNFIAPE
YAYKIVKKGDSTIMKSEVEYGNCNTKCQTPIGAINSSMPFHNIHPLTIGECPKYVKSNKLVLATGLRNSPQNVPQRETRGLFGAIAGFIE
GGWQGMVDGWYGYHHSNEQGSGYAADKESTQKAIDGITNKVNSIIDKMNTQFEAVGKEFNNLERRIENLNKKMEDGFLDVWTYNA
ELLVLMENERTLDFHDSNVKNLYDKVRLQLRDNAKELGNGCFEFYHKCDNECMESVRNGTYDYPQYSEEARLNREEISGVKLESMG
TYQILSIYSTVASSLALAIMIAGLSFWMCSNGSLQCRICI

IAN5

MEKIVLLLAIVSLVKSDQICIGYHANNSTEQVDTIMEKNVTVTHAQDILEKTHNGKLCSLNGVKPLILKDCSVAGWLLGNPMCDEFLNVP
EWSYIVEKDNPANGLCYPGNFNDYEELKHLLSSTNHFEKIQIIPRSSWSNHDASSGVSSACPYNGRSSFFRNVVWLIKKNNAYPTIKR
SYNNTNQEDLLILWGIHHPNDAAEQTKLYQNPTTYVSVGTSTLNQRSVPEIATRPKVNGQSGRMEFFWTILKPNDAINFESNGNFIAP
EYAYKIVKKGDSAIMKSELEYGNCNTKCQTPVGAINSSMPFHNIHPLTIGECPKYVKSNKLVLATGLRNIPQNSPQRERRRKRGLFGAI
AGFIEGGWQGMVDGWYGYHHSNEQGSGYAADKESTQKAIDGVTNKVNSIIDKMNTQFEAVGREFNNLERRIENLNKKMEDGFLDV
WTYNAELLVLMENERTLDFHDSNVKNLYDKVRLQLRDNAKELGNGCFEFYHKCDNECMESVRNGTYDYPQYSEEARLKREEISGVK
LESIGTYQILSIYSTVASSLALAIMVAGLSLWMCSNGSLQCRICI

IAN6

MEKIVLLFATISLVKSDQICIGYHANNSTEQVDTIMEKNVTVTHAQDILEKTHNGKLCDLNGVKPLILKDCSVAGWLLGNPMCDEFIRVP
EWSYIVERANPANDLCYPGSLNDYEELKHLLSRINHFEKTLIIPKSSWPNHETSLGVSAACPYQGTPSFFRNVVWLIKKNDAYPTIKISY
NNTNREDLLILWGIHHSNNAAEQTNLYKNPTTYVSVGTSTLNQRLVPKIATRSQVNGQRGRMDFFWTILKPNDAIHFESNGNFIAPEY
AYKIVKKGDSTIMKSEMEYGHCNTKCQTPIGAINSSMPFHNIHPLTIGECPKYVKSNKLVLATGLRNSPLNSPQRERRRKRGLFGAIAG
FIEGGWQGMVDGWYGYHHSNEQGSGYAADKESTQKAIDGVTNKVNSIIDKMNTQFEAVGREFNNLERRIENLNKKMEDGFLDVWT
YNAELLVLMENERTLDFHDSNVKNLYDKVRLQLRDNAKELGNGCFEFYHKCDNECMESVRNGTYDYPQYSEEARLKREEISGVKLE
SIGTYQILSIYSTVASSLALAIMVAGLSLWMCSNGSLQCRICI

IAN7

MENIVLLLAIVSLVKSDQICIGYHANNSTEQVDTIMEKNVTVTHAQDILEKTHNGKLCDLNGVKPLILKDCSVAGWLLGNPMCDEFIRVP
EWSYIVERANPANDLCYPGNLNDYEELKHLLSRINHFEKILIIPKSSWPNHETSLGVSAACPYQGTPSFFRNVVWLIKKNDAYPTIKISY
NNTNREDLLILWGIHHSNNAAEQTNLYKNPTTYISVGTSTLNQRLVPKIATRSQVNGQRGRMDFFWTILKPNDAIHFESNGNFIAPEYA
YKIVKKGDSTIMKSEVEYGHCNTKCQTPIGAINSSMPFHNIHPLTIGECPKYVKSNKLVLATGLRNSPLNSPQRERRRKRGLFGAIAGFI
EGGWQGMVDGWYGYHHSNEQGSGYAADKESTQKAIDGVTNKVNSIIDKMNTQFEAVGREFNNLERRIENLNKKMEDGFLDVWTYN
AELLVLMENERTLDFHDSNVKNLYDKVRLQLKDNAKELGNGCFEFYHKCNNECMESVRNGTYDYPQYSEEARLKREEISGVKLESIG
IYQILSIYSTVASSLVLAIMMAGLSLWMCSNGSLQCRICI

IAN8

MEKIVLLFAIVSLVKSDQICIGYHANNSTEQVDTIMEKNVTVTHAQDILEKTHNGKLCDLNGVKPLILKDCSVAGWLLGNPMCDEFINVP
EWSYIVEKANPANDLCYPGNFNDYEELKHLLSRINHFEKIQIIPKSSWSDHEASLGVSAACPYQGNSSFFRNVVWLIKKNNAYPTIKKS
YNNTNQEDLLVLWGIHHPNDAAEQTRLYQNPTTYISVGTSTLNQRLVPKIATRSKVNGQSGRMDFFWTILKPNDAIHFESNGNFIAPE
YAYKIVKKGDSTIMKSELEYGNCNTKCQTPIGAINSSMPFHNIHPLTIGECPKYVKSNKLVLATGLRNSPQNSPQRERRRKRGLFGAIA
GFIEGGWQGMVDGWYGYHHSNEQGSGYAADKESTQKAIDGVTNKVNSIIDKMNTQFEAVGREFNNLERRIENLNKKMEDGFLDVW
TYNAELLVLMENERTLDFHDSNVKNLYDKVRLQLKDNAKELGNGCFEFYHKCNNECMESVRNGTYDYPQYSEEARLKREEISGVKL
ESIGIYQILSIYSTVASSLVLAIMMAGLSLWMCSNGSLQCRICI

Figure S1. Amino Acid sequences represented from each vaccine. Leader sequence is highlighted in light grey, HA1 sequence in white, HA2 sequence in dark grey.

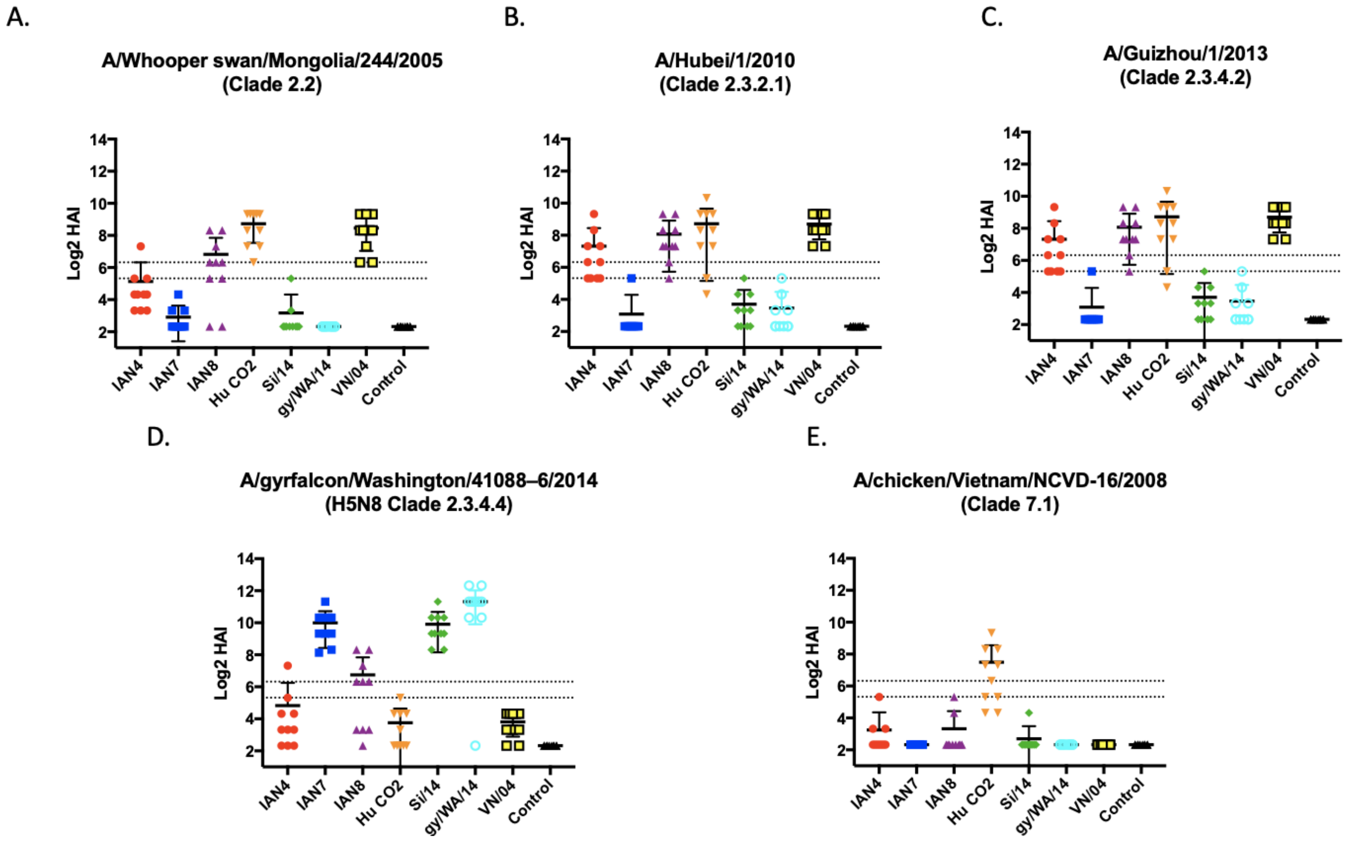

Figure S2: Virus HAI antibody panel from next generation COBRA vaccines. Five viruses from 4 viral clades were chosen to represent clades that are currently circulating around the globe in wild waterfowl populations. The virus panel is arranged from newest virus (2014) to the oldest (2005). The viral clades include 2.3.4.4, 2.3.2.1, 2.3.4.2 and 2.2 respectively. IAN-4, IAN-7 and IAN-8 were chosen to continue with further studies. Other vaccines were excluded due to their similar breath to wildtype vaccines or lack of immunogenicity.

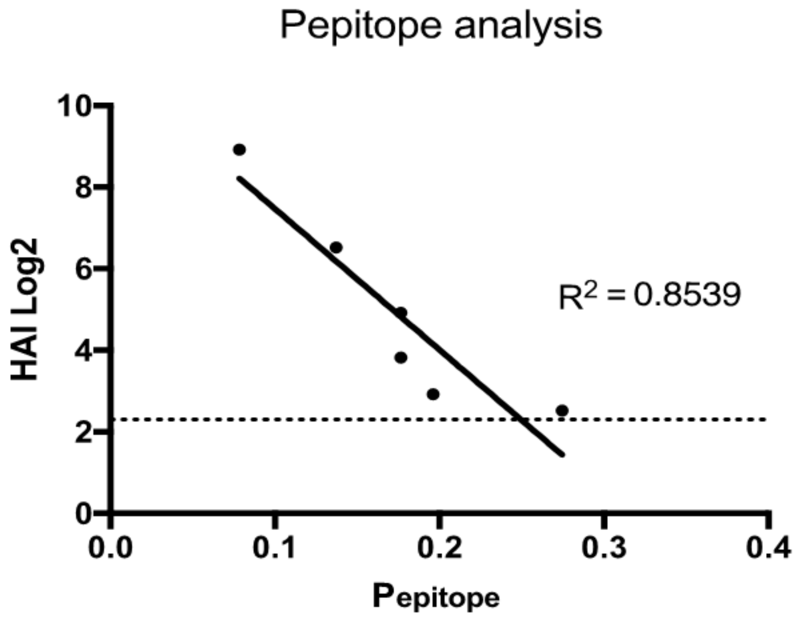

$$P_{sequence} = \frac{\textbf{Number of substitiutions in the HA1 RBS domain of hemagglutinin}}{\textbf{Total number of amino acids in the HA1 RBS domain of hemagglutinin}}$$

Figure S3. Pepitope analysis of HAI data plotted against amino acid sequence similarity. There were 51 amino acids total analyzed in the HA1 portion of the H5 molecule. Amino acid sequences were aligned and analyzed using Geneious® software. the number of amino acid differences was divided by the total amino acid epitopes associated with the RBS. Linear regression analysis was performed on all strains of virus and vaccine HAI titer, there was a significant correlation between HAI titer and Psequence.

Vaccine	Sequences used
IAN-1	Traditional COBRA 2013-2017
IAN-2	Rolling COBRA 2011-2015
IAN-3	Rolling COBRA 2011-2015
IAN-4	Rolling COBRA 2012-2016
IAN-5	Rolling COBRA 2011-2015
IAN-6	Rolling COBRA 2012-2016
IAN-7	Rolling COBRA 2014-2018
IAN-8	Rolling COBRA 2011-2016

Table S1: Table of recombinant HA proteins used to vaccinate mice in preliminary serological study. BALB/c female mice were vaccinated on a prime-boost-boost regimen with soluble recombinant HA protein in combination with an oil-in-water emulsion Addavax adjuvant. COBRA vaccines termed IAN-1 – IAN-8 were used along with wild-type comparators to compare the antibodies elicited in a HAI panel.
